# Progressive Developments from 1090 Onwards

**Published:** 1918-07-27

**Authors:** 


### THE HISTORY OF THE ORDER OF ST. JOHN.*

### Progressive Developments from 1090 Onwards.

WHEN Malta was taken by the Normans in 1090 it was found that a few merchants of Amalfi, trading with the Levant, had established in Jerusalem, with the consent of the Caliph of Egypt, a house for the purpose of affording relief and alleviating the injuries and the suffering of Christian pilgrims on their journey to the Holy City. This afterwards became a hospital for the reception of pilgrims, who, as *hospites* or guests, were entertained and succoured on their weary way—for they generally arrived robbed and plundered as well as injured and sick; their hosts in Jerusalem being described as hospitallers. There was also a house of ladies belonging to this Order, where women gave their services to the needs of pilgrims and wayfarers of their own sex. The Order was established about this period, and 1092 is given as the actual date, but it extended its benevolent work of ministration to the sick into this country, and in 1100 began to found the ancient Priory of Clerkenwell. It had a useful although a chequered career; it flourished in England for over two hundred years, but it migrated from Jerusalem when the Holy City was taken from the Christians and moved into Rhodes in 1310 and to Malta from Rhodes in 1522. It was suppressed in England in 1540, when its property was confiscated and its riches despoiled. In 1547 it was restored by Royal Charter, but in 1559 its possessions were again confiscated and the Order once more suppressed, most of the Brethren returning to the headquarters in Malta; but the loss of this island in 1798 at the outset of the Napoleonic expedition into Egypt ended its good work for a generation, and only since 1830—when the Order was again revived in England—has its great usefulness in this country started afresh. Through the personal support and munificence of Sir Edmund A. H. Lechmere, the Order in 1874 was once more housed in a part of its original monastic foundation at St. John's Gate. In 1877 the St. John Ambulance Association was founded and established, firstly, to organise a civilian reserve for the Army Medical Service, and, secondly, for the dissemination of instruction in "first aid," which meant the early preliminary treatment of the sick and injured pending the arrival of the doctor, and for the teaching of women in the first principles of home nursing and hygiene. In 1882 was started an additional branch of its useful work, which endeavoured to provide trained help through the St. John Ambulance Brigade in public places, crowded streets, or at great popular gatherings. This section of its work encouraged the deposit in appropriate localities of stretchers, splints, dressings, bandages, and surgical appliances, to be used as first aid in case of accidents, and this brigade work has concerned itself especially with the development of ambulance corps to provide a safe transport for the sick and wounded not only during peace time in our crowded streets, but also in time of war, as auxiliary units for the medical services of the Navy and Army. In 1888 the Order was incorporated for the second time under Royal Charter, with his Majesty the King as Sovereign-head and patron. The ambulance section has issued special text-books, of which over half a million have been sold, and the "First Aid" Guide, brought out originally by Sir James Cantlie, is in its thirty-third edition, which demonstrates its usefulness and its appreciation.

Many thousands of detached classes have been organised in hundreds of administrative centres throughout the United Kingdom, India, Australia, Canada, and South Africa. Since the war this great work has almost incredibly extended in every direction. Last year the Ambulance Section granted over 83,000 certificates after personal examination in the practical work of relief; its brigade of voluntary workers for Army Medical Service numbers over 65,000 men and women, out of which over 22,000 have been called up for Army and Navy Reserve duties, whilst 9,000 additional men are engaged in ambulance work, and over 15,000 nursing sisters are enrolled in V.A.D. work. The great traditions of the founders of the Hospitaller Knights have been completely fulfilled by the Order, and since its last incorporation by Royal Charter it has not only taught but it has practised the high aims and the great ideals of the Hospitaller Brethren. The Order at the present time has 234 auxiliary hospitals under its auspices, whilst those under the Joint Committee of the Order and the Red Cross number 1,448 hospitals, some of them being great mental hospitals, and together they provide beds for 81,514 patients. The Order, which to-day so justly merits public approval and so richly justifies public confidence, has since October 1914 been intimately associated with the British Red Cross Society—formerly the British National Aid Society—and a few words must be devoted to their signs and symbols. Its white cross on scarlet—its military knightly emblem—stands side by side with the English Red Cross of St. George, also upon white, and this College is an outward and visible embodiment of purpose and effort in the relief of human suffering.

The Cross is the common sign and symbol of the united aims of these two great institutions which bear help and carry relief to the sick and wounded; it still remains, as in the days of old, the symbol of the highest dignity and honour. As in the past, when the Cross indicated the noblest insignia of knightly estimation of rank, so to-day it forms the proudest ensign upon the Royal diadem, and it continues to be the emblem of help in distress, of ease from pain, and of relief from suffering It is the symbol of succour in distress and of comfort in the direst need, and under its flag are mitigated the distress and torture which are inevitable from the horrors of war. Justin Martyr said that the sign of the Cross was impressed on the whole of Nature. Hardly a handicraftsman but uses its figure among the implements of his industry. It forms a part of man himself, as may be seen when he raises his hands in prayer. Mediæval artists rivalled in designing it for decoration and enrichment, and no inscription in the Middle Ages began without it. It adorns the crowned sceptre of princes, and the greatest warriors of old were proud to see it upon the hilts of their swords and on the banners under which they fought. In private life it forms the most beautiful and highly prized personal ornament, and it continues to denote the profession and rank of the highest ecclesiastical dignitaries. It is even used to-day to imply the duties to the State which are incumbent upon every earner of his daily bread—viz., to save money—for every advertisement and poster inviting the purchase of War Certificates has that most singular and ancient modification of the Cross, formerly so much in favour as an emblem of secret societies, and described as the "Fylfot," the Cross of Thor, or the *gammadion*, placed as a sign and an earnest invitation to contribute towards victory. The four equal arms, each with its rectangular spur, must be a familiar object to those who travel in tramcars and motor 'buses, for it is seen everywhere as an emblem of active help and self-denial.

